# Lysine pyruvylation: decoding the molecular language of pyruvate signaling

**DOI:** 10.1038/s44321-026-00522-5

**Published:** 2026-09-16

**Authors:** Jun Zhang

**Affiliations:** Department of Biochemistry and Molecular Biology, Guangdong Key Laboratory of Genome Instability and Human Disease Prevention, Shenzhen University Medical School, Shenzhen, China

**Keywords:** Metabolism

## Abstract

In this Comment article, Jun Zhang discusses the emerging role of lysine pyruvylation as a metabolic regulator and outlines the key evidence needed to establish its relevance as a biomarker and therapeutic target in human disease.

**Figure:**
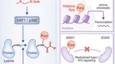

## Teaser

Lysine pyruvylation (Kpy) converts pyruvate availability into a reversible protein modification, placing a central carbon metabolite directly within cellular regulatory circuits. An initial study identified STAT1 K201 pyruvylation and showed that this modification suppresses type I interferon signaling by disrupting STAT1–STAT2 assembly. A subsequent study established HAT1 and p300 as Kpy writers and SIRT3 as a Kpy eraser, linking histone Kpy to glycolytic flux, active chromatin and transcription. Together, these findings introduce Kpy as a potential interface between metabolism, chromatin and immunity. However, evidence is currently limited to two initial mechanistic studies: disease causality, abundance and occupancy in human tissues, and translational utility remain unestablished. This commentary therefore distinguishes demonstrated mechanisms from testable hypotheses and outlines the evidence required before Kpy can be considered a disease biomarker or therapeutic target.

## A metabolic modification enters physiology

Metabolic state is increasingly understood as regulatory information encoded through protein chemistry. Recent reports on acetylation, lactylation, and other metabolite-linked lysine acylations have shown how nutrient availability can influence chromatin, signaling, and cell fate (Chen et al, 2024; Nitsch et al, 2021; Sabari et al, 2016; Sahu and Lu, 2025; Sheng et al, 2026; Zhang and Schroeder, 2025; Zhang et al, 2019). These precedents make pyruvate especially intriguing. As the terminal product of glycolysis and a branch point between mitochondrial oxidation, lactate production, and biosynthesis, pyruvate is positioned to report metabolic flux. Yet pyruvate has generally been viewed as a substrate or allosteric regulator rather than a covalent signaling input. The discovery of Kpy changes this view by suggesting that pyruvate-derived chemistry can be written onto proteins and interpreted through changes in protein function (Song et al, 2026; Zuo et al, 2026).

Two complementary studies establish the mechanistic foundation of this concept. The initial study identified STAT1 K201 pyruvylation and showed that it disrupts STAT1–STAT2 assembly, thereby restraining type I interferon signaling (Zuo et al, 2026). A subsequent study demonstrated that HAT1- and p300-dependent histone Kpy responds to glycolytic flux and associates with active promoters and transcription, while SIRT3 removes Kpy (Song et al, 2026). Thus, the currently established branches should be kept distinct: STAT1 Kpy has a demonstrated immune-signaling function, whereas the identified HAT1/p300–SIRT3 machinery has been linked to histone Kpy and chromatin regulation. Whether the same enzymes regulate STAT1 Kpy has not been shown.

The significance of Kpy, however, will not be determined by how many modified proteins can be cataloged. The critical question is whether endogenous Kpy reaches functionally meaningful occupancy at the right substrate, location, and time to control physiology. Pyruvate abundance alone cannot answer this question because changes in pyruvate also reshape redox balance, lactate production, acetyl-CoA supply, and mitochondrial flux. Demonstrating causality will therefore require separating Kpy from the broader metabolic consequences of its precursor.

## A context-dependent metabolic checkpoint

Kpy is most likely to become influential when pyruvate changes rapidly, and cells must coordinate transcription with stress signaling. This principle highlights three candidate settings—cancer, immunity, and tissue adaptation—while distinguishing plausible roles from established functions.

Cancer provides a stringent test context because tumors combine elevated glycolytic flux, heterogeneous oxygenation, altered mitochondrial pyruvate handling, and chromatin plasticity (Demicco et al, 2024; Faubert et al, 2020; Fendt et al, 2020; Finley, 2023; Hanahan, 2022; Martínez-Reyes and Chandel, 2021). Histone Kpy could convert these metabolic conditions into transcriptional programs that support cell-state switching, stress tolerance, or treatment persistence. Non-histone Kpy may further influence transcription factors, DNA repair, or metabolic enzymes. Yet bulk pyruvate or pan-Kpy staining will be insufficient: pyruvate is dynamically consumed, exported, and compartmentalized, and writer availability may constrain modification. Spatially resolved measurements of pyruvate, pyruvyl-CoA, and site-specific Kpy across tumor, stromal, and immune compartments are needed to determine whether Kpy actively drives tumor adaptation or merely accompanies it.

Immunity offers the clearest functional entry point. STAT1 pyruvylation directly links metabolic state to the threshold of type I interferon signaling (Zuo et al, 2026). Transient Kpy may limit tissue damage after pathogen control, whereas sustained Kpy could enable viral persistence or weaken antitumor immunity. This duality has immediate translational implications: suppressing STAT1 Kpy might strengthen antiviral or antitumor responses but also exacerbate immunopathology, while promoting it could restrain interferonopathies at the cost of host defense. Kpy should therefore be viewed as a context-dependent immune rheostat rather than a uniformly beneficial or harmful modification.

Aging, regeneration, and metabolic disease provide a broader frontier because they combine mitochondrial dysfunction, inflammatory remodeling, and epigenetic instability (Baker and Rutter, 2023; Haws et al, 2020; Martínez-Reyes and Chandel, 2020). At present, however, there is no direct evidence that Kpy contributes to any of these conditions. Their relevance should be evaluated through tissue-resolved Kpy measurements and causal site-specific models rather than inferred from pyruvate abundance alone.

## From mechanism to translation

It is premature to position Kpy as a biomarker because its endogenous abundance, chemical stability, tissue distribution, dynamic range, and association with human disease remain undefined. Kpy may be scarce, chemically labile, or confined to specific substrates and subcellular compartments. The immediate priority should therefore be rigorous analytical validation rather than clinical deployment. Candidate signals must be distinguished from structurally related lysine acylations, particularly acetylation and lactylation, using orthogonal mass spectrometry, isotope tracing, competition controls, synthetic standards and, where possible, site-occupancy measurements (Aebersold and Mann, 2016; Jang et al, 2018; Qin et al, 2023; Sahu and Lu, 2025). These approaches should establish assay specificity, sensitivity, and detection limits and determine whether site-specific Kpy can be reproducibly quantified in accessible specimens. Biomarker development should be considered only if Kpy provides diagnostic or pharmacodynamic information beyond that conveyed by pyruvate, lactate, and relevant pathway proteins.

**Figure 1:**
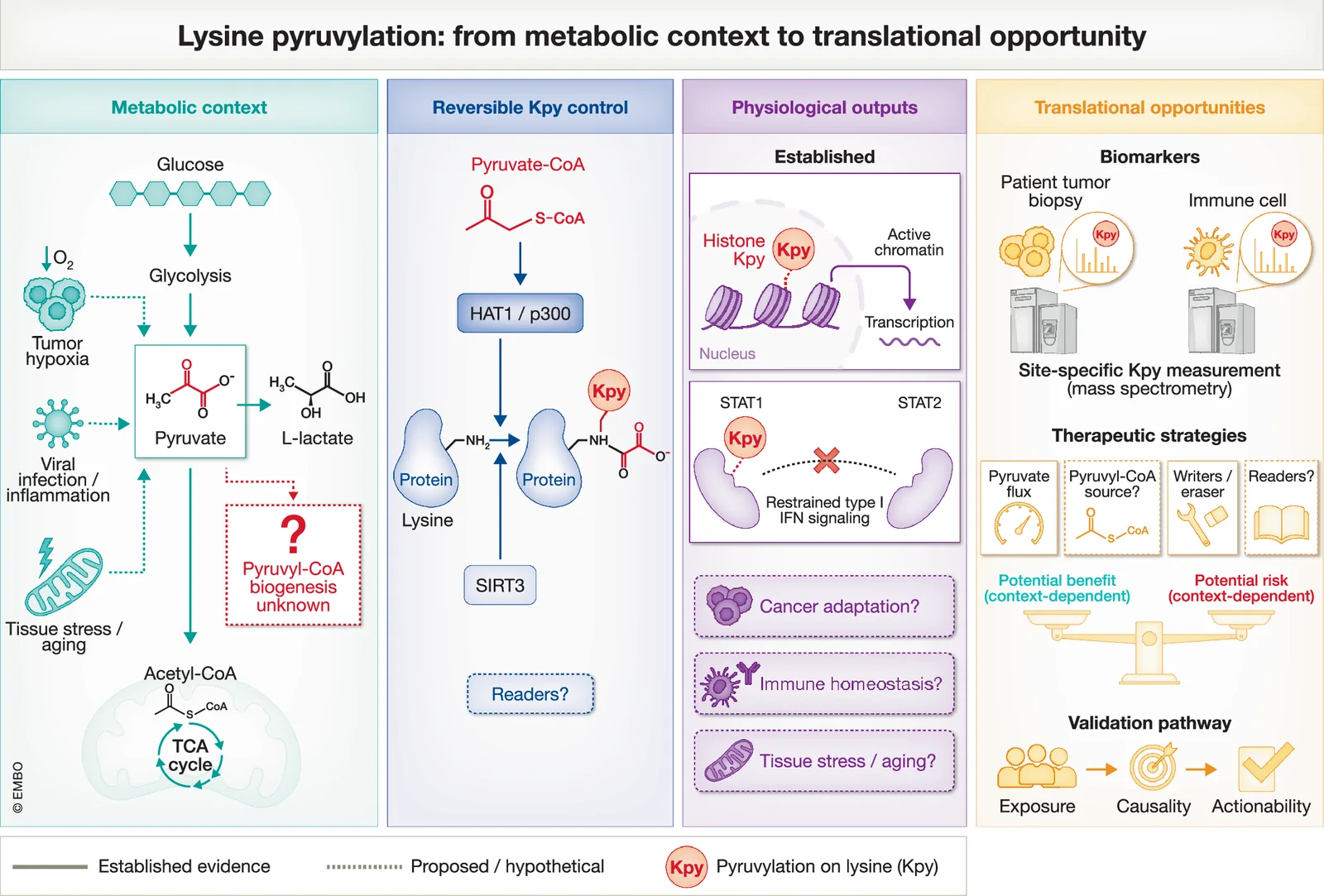
Lysine pyruvylation: established mechanisms and priorities for validation. L-Lactate and pyruvate sit at a central metabolic branch point. Pyruvate-derived chemistry supports Kpy, although the endogenous source of pyruvyl-CoA remains unresolved. HAT1 and p300 catalyze histone Kpy, and SIRT3 removes Kpy, whereas STAT1 K201 pyruvylation disrupts STAT1–STAT2 assembly and restrains type I interferon signaling; the enzyme(s) responsible for STAT1 Kpy have not been established. Broader roles in cancer, immune homeostasis, tissue stress, and aging are hypotheses. Because Kpy abundance, occupancy, and disease associations remain undefined, translational development should proceed through quantitative exposure measurement, Kpy-selective enzymology, site-specific causality, and disease validation before biomarker qualification or drug discovery. Solid lines indicate established evidence; dashed lines indicate proposed or hypothetical relationships.

Beyond its potential biomarker value, the Kpy axis presents four putative therapeutic intervention points: pyruvate flux, pyruvyl-CoA production, Kpy writers or erasers, and future reader proteins (Fig. 1). Manipulating glycolysis, lactate dehydrogenase, or mitochondrial pyruvate handling is feasible but pleiotropic, affecting energy production, redox balance, and multiple metabolite-derived modifications (Demicco et al, 2024; Finley, 2023; Martínez-Reyes and Chandel, 2021). HAT1, p300, and SIRT3 are more proximal, yet none is dedicated to Kpy; targeting them will inevitably alter additional acylations and biological programs.

Greater selectivity may emerge from two unresolved components of the pathway. Identifying the enzymatic source of pyruvyl-CoA could reveal a conditionally active upstream target, whereas bona fide Kpy readers might allow pathogenic signal interpretation to be blocked without globally eliminating the modification. Both possibilities remain speculative until pyruvyl-CoA formation is directly traced and reader recognition is demonstrated biochemically and structurally, with endogenous genetic rescue establishing functional causality. Ultimately, the therapeutic consequences of targeting Kpy are likely to be cell- and disease-specific. Inhibiting STAT1 Kpy, for example, may restore type I interferon signaling, whereas limiting histone Kpy could suppress adaptive transcriptional programs in tumors. Because these effects may coexist or even oppose one another within the same tissue, substrate selectivity, targeted delivery, transient dosing, and pharmacodynamic monitoring will be essential for translating Kpy biology into a viable therapeutic strategy.

## A roadmap from signal to action

Four milestones can determine whether Kpy becomes biologically and eventually translationally actionable. First, establish exposure by quantifying pyruvate, pyruvyl-CoA, and site-specific Kpy in cells and human tissues with spatial and temporal resolution. Second, establish enzymology by identifying Kpy-selective transferases, erasers, and readers and defining their donor chemistry. Third, establish causality using lysine-site mutants, catalytic separation-of-function alleles, and lineage-restricted models to show that Kpy itself—not parallel activities of its regulators—controls a phenotype. Fourth, establish disease relevance by demonstrating reproducible alteration and causal contribution in clinically representative models. Biomarker qualification and drug discovery should be considered only after these criteria are met.

Kpy is conceptually important because it connects a central metabolic branch point to chromatin regulation and interferon competence. Its broader physiological and disease relevance, however, remains unknown. Current evidence does not justify Kpy as a clinical biomarker or therapeutic target, particularly before endogenous abundance, tissue distribution, and causal disease functions are established. The field should therefore prioritize quantitative validation, selective enzymology and site-specific genetics. These experiments will determine whether Kpy is a restricted regulatory event, a broader metabolic language, or both.

## Lessons from lysine lactylation

The evolution of the lysine lactylation field offers an instructive framework for resolving Kpy enzymology. Although p300 and sirtuins were initially implicated as broad regulators of lysine lactylation, subsequent studies identified AARS1 and AARS2 as lactate-responsive lactyltransferases with distinct cytosolic/nuclear and mitochondrial functions (Ju et al, 2024; Li et al, 2024; Mao et al, 2024; Zong et al, 2024). This progression illustrates that enzymes capable of installing or removing a modification experimentally may not represent the complete or most selective regulatory machinery.

By analogy, HAT1 and p300 may reveal the feasibility of Kpy formation without exhausting the repertoire of physiologically relevant Kpy enzymes. Future screens should therefore extend beyond canonical acyltransferases to metabolic enzymes and aminoacyl-tRNA synthetases, testing both ATP- and acyl-CoA-dependent mechanisms of pyruvyl transfer. Candidate enzymes should be validated using purified components, isotope-defined donors, catalytic mutants, and genetic rescue experiments that distinguish Kpy-dependent activity from their canonical functions. Identifying a Kpy-selective transferase would substantially strengthen causal studies and potentially provide a more precise therapeutic entry point; however, no such enzyme has yet been established.

## Supplementary information

Peer Review File
