# Supplementary material for: Lysine pyruvylation: decoding the molecular language of pyruvate signaling
Source: EMBO Mol Med. 2026 Sep 16;18(10):3879–82. doi: 10.1038/s44321-026-00522-5 (PMC13658220; doi:10.1038/s44321-026-00522-5)
Supplement: Supplementary file 1 [file 44321_2026_522_MOESM1_ESM.pdf]

EMBO
Molecular Medicine

Lysine pyruvylation: decoding the molecular language of pyruvate signaling

Jun Zhang

Corresponding author: Jun Zhang (junzh128@szu.edu.cn)

Review Timeline:	Submission Date:	23rd Jul 26
	Editorial Decision:	13th Aug 26
	Revision Received:	16th Aug 26
	Accepted:	18th Aug 26

Editor: Zeljko Durdevic

Transaction Report:

(Note: With the exception of the correction of typographical or spelling errors that could be a source of ambiguity, letters and reports are not edited. Depending on transfer agreements, referee reports obtained elsewhere may or may not be included in this compilation. Referee reports are anonymous unless the Referee chooses to sign their reports.)

13th Aug 2026

Dear Dr. Zhang,

Thank you for the submission of your manuscript to EMBO Molecular Medicine. We have now received feedback from the two reviewers who agreed to evaluate your manuscript. As you will see from the reports below, the referees are positive about its interest and timeliness, however, they also raise serious criticisms that should be fully addressed in a major revision of the current manuscript.

I would also like to ask you to amend the following:
1) Upon editorial assessment we may send the article to our graphic artist who will re-draw the figures for style and clarity. Therefore, please remove the figure from the manuscript file and submit it as individual high-resolution files in an editable format (e.g. PDF with the option 'editable labels' checked).
2) Figure legends should be placed at the end of the manuscript text.
3) Please enter the complete funding information in our submission system.
4) Rename "Competing Interests" to "Disclosure and competing interests statement". We updated our journal's competing interests policy in January 2022 and request authors to consider both actual and perceived competing interests. Please review the policy https://www.embopress.org/competing-interests and update your competing interests if necessary.
5) Please correct the reference citation in the reference list. Where there are more than 10 authors on a paper, 10 will be listed, followed by "et al.". Please check "Author Guidelines" for more information.
https://www.embopress.org/page/journal/17574684/authorguide#referencesformat
6) Please provide a point-by-point letter INCLUDING my comments as well as the reviewer's reports and your detailed responses (as Word file).

I hope that the referees' comments do not prove too problematic to address and I look forward to reading your next version.

Yours sincerely,

Zeljko Durdevic

Zeljko Durdevic
Senior Editor
EMBO Molecular Medicine

*** IMPORTANT INFORMATION ***

When submitting your revised manuscript, please include:

1) a .doc formatted version of the manuscript text (including Figure legends and tables)
2) Separate figure files
3) a letter INCLUDING the reviewer's reports and your detailed responses to their comments.

Also, and to save some time should your paper be accepted, please read below for additional information regarding some features of our research articles:

1) Glossary: EMBO Molecular Medicine articles will be accompanied by a glossary explaining some of the terms used for laymen. I identified the following:

_____, _____, _____

Could you please help us in identifying terms that may need an "explanation" other terms that we can add to the glossary.

2) For more information: This is a short list of related web links for further consultation by the readers. Could you identify some relevant ones? Examples are patient associations, OMIM related links, databases, authors websites, etc.

3) Pending issues: At the end of each article we will have a box highlighting issues that still need further studies and where research efforts should converge (we call this the Pending issues box). From my reading I would say:

----

but I can see there may be many more. Could you work on this as well?

4) Disclosure and competing interest statement: Please include a statement declaring any competing commercial interests in relation to your submitted work.

5) Please note that we now mandate that all corresponding authors list an ORCID digital identifier. This takes <90 seconds to complete. We encourage all authors to supply an ORCID identifier, which will be linked to their name for unambiguous name identification.

Currently, our records indicate that the ORCID for your account is 0000-0002-6523-7573.

Please click the link below to modify this ORCID:
Link Not Available
-

Thank you,

Zeljko Durdevic

Zeljko Durdevic
Editor
EMBO Molecular Medicine

***** Reviewer's comments *****

Referee #1 (Remarks for Author):

This is a nice, timely article by Dr. Jun zhang. In general, this paper is well written and should be of interest to many readers. However, several key issues remain to be addressed:

1. It remain unknown if Kpy pathway is important to cause a disease. I think it is too early to discuss biomarkers. We do not know if and how this pathway is associated with diseases and contribute to disease progression. Kpy is likely present in low abundance and not suitable for biomarker.
2. Likelywise, we do not know if this pathway contributes to the diseases. We have too little information. it is therefore premature to provide perspective for drug discovery based on this pathway.
3. In Figure 1, lactate should be labeled as L-lactate. In addition, its structure should be written to show its isomer.
4. Many references are not adequately cited and should be carefully checked. Some examples are suggested below: i) The first paragraph: some of the references ((Chen, He etal., 2024, Choudhary, Weinert et al., 2014, Kaelin & McKnight, 2013, Sabari, Zhang et al., 2016, Zhang & Schroeder, 2025, Zhang, Tang et al., 2019)) are too old and should be removed, including those before 2015. For lactylation review, this paper should be added: DOI: 10.1038/s41580-025-00876-7

Referee #2 (Remarks for Author):

This manuscript presents a very recent finding-protein pyruvylation, highlighting an attractive new field that potentially pushes forward the development of both protein post-translational modifications and signaling transduction. I have several comments for consideration.
1. The description "Initial studies identify HAT1 and p300 as pyruvyltransferases and SIRT3 as a depyruvylase, and connect Kpy to active chromatin and suppression of STAT1-dependent interferon signaling." is not accurate. There is no evidence showing

that either HAT1/p300 or SIRT3 contributes to suppression of STAT1-dependent interferon signaling. In addition, the initial study in this field is not about HAT1 and p300 as a pyryvyltransferases, but the initial identification of protein pyruvylation. Based on the actual process of this research about protein pyruvylation, this description should be revised to "An initial study identified lysine pyruvylation on STAT1 proteins and revealed STAT1 pyrvuvylation can suppress interferon signaling, and a subsequent study demonstrated HAT1 and p300 as pyruvyltransferases and SIRT3 as a depyruvylase, which connect Kpy to active chromatin. "

2. Accordingly, this sentence "HAT1-dependent histone Kpy couples glycolytic flux to active promoters and transcription, whereas p300-mediated STAT1 pyruvylation disrupts STAT1-STAT2 assembly and restrains type I interferon signaling" should be revised. There is no data showing that p300 could mediate STAT1 pyruvylation and regulate STAT1-STAT2 assembly. It should be revised to "STAT1 pyruvylation disrupts STAT1-STAT2 assembly and restrains type I interferon signaling, whereas p300/HAT1-dependent histone Kpy couples glycolytic flux to active promoters and transcription."

3. The author mentioned "Transient Kpy may limit tissue damage after pathogen control". Can the author explain how this hypothesis was concluded? Any references?

4. As shown in lactylation studies, the relative specific transferases for lactylation, including AARS1 and AARS2, were identified although HAT/p300 and SIRT are firstly shown to be able to regulate lactylation. Can the author discuss whether there are also specific transferases for pyruvylation? In my opinion, this specific pyruvyltransferases could be important for future pyruvylation studies.

Referee #1

Comment: 1. It remain unknown if Kpy pathway is important to cause a disease. I think it is too early to discuss biomarkers. We do not know if and how this pathway is associated with diseases

and contribute to disease progression. Kpy is likely present in low abundance and not suitable for biomarker.

Response: Thank you very much for the comment. We agree. We have removed the affirmative biomarker framing and explicitly state that no study has established Kpy distribution, stability, dynamic range, disease association or clinical utility. The revised text highlights that low abundance, chemical lability and restricted substrate/compartment distribution may preclude robust tissue measurement. It now defines orthogonal mass spectrometry, isotope tracing, synthetic standards, competition controls, detection limits and site-occupancy measurements as prerequisites. Biomarker development is presented only as a future possibility contingent on reproducible measurement and added information beyond pyruvate, lactate and pathway-protein abundance.

Comment: 2. Likelywise, we do not know if this pathway contributes to the diseases. We have too little information. it is therefore premature to provide perspective for drug discovery based on this pathway.

Response: Thank you very much for the comment. The revised manuscript now states directly that Kpy is not a validated drug-discovery axis. We removed the former list of four intervention opportunities and emphasize the pleiotropy of perturbing glycolysis, LDH, mitochondrial pyruvate transport, HAT1, p300 or SIRT3. Therapeutic work is deferred until a defined Kpy event is shown to causally contribute to disease, to be selectively modulated at tolerable exposure, and to yield benefit separable from other functions of the targeted enzyme.

Comment: 3. In Figure 1, lactate should be labeled L-lactate and its structure should show the isomer.

Response: Thank you very much for the constructive suggestion. Figure 1 now labels the metabolite as "L-lactate" and adds the (S) stereochemical designation. The figure legend has also been revised to use L-lactate.

Comment: 4. References are not adequately cited; remove older citations from the first paragraph, including pre-2015 references, and add DOI 10.1038/s41580-025-00876-7.

Response: We revised the opening paragraph to rely on recent, directly relevant work and removed its pre-2015 citations. The requested Nature Reviews Molecular Cell Biology review by Sheng et al. has been included and is cited where analytical overlap with L-lactylation is discussed.

Referee #2

Comment: 1. Correct the inaccurate description of the initial studies and do not imply that HAT1/p300 or SIRT3 suppress STAT1-dependent interferon signaling.

Response: Thank you very much for the comment. We agree and have adopted the reviewer's chronological and mechanistic distinction in both the Abstract and main text. The revised text states

that the initial study identified STAT1 K201 pyruvylation and showed suppression of type I interferon signaling through disruption of STAT1–STAT2 assembly. It then states that the subsequent study identified HAT1 and p300 as Kpy writers and SIRT3 as a Kpy eraser and linked this machinery to histone Kpy, active chromatin and transcription. We explicitly note that HAT1/p300/SIRT3 regulation of STAT1 Kpy has not been demonstrated.

Comment: 2. Revise the sentence assigning p300-mediated STAT1 pyruvylation and STAT1–STAT2 disruption.

Response: Thank you very much for the comment. The revised sentence reads: “The initial study identified STAT1 K201 pyruvylation and showed that it disrupts STAT1–STAT2 assembly, thereby restraining type I interferon signaling. A subsequent study demonstrated that HAT1- and p300-dependent histone Kpy responds to glycolytic flux and associates with active promoters and transcription, while SIRT3 removes Kpy.”

Comment: 3. The author mentioned "Transient Kpy may limit tissue damage after pathogen control". Can the author explain how this hypothesis was concluded? Any references?

Response: We agree that this statement exceeded the available evidence. Rather than defend an unsupported hypothesis, we removed it. The revised paragraph confines the conclusion to the demonstrated attenuation of antiviral interferon signaling and identifies tissue protection versus viral persistence as alternative outcomes that require direct testing.

Comment: 4. Discuss whether specific pyruvyltransferases may exist, analogous to AARS1 and AARS2 in lactylation.

Response: Thank you very much for the constructive suggestion. We added a dedicated paragraph using AARS1 and AARS2 as an instructive precedent. The text now proposes that HAT1/p300 may reveal Kpy chemistry without exhausting its enzymology and recommends systematic testing of metabolic enzymes and aminoacyl-tRNA synthetases using purified-component assays, isotope-defined donors, ATP/acyl-CoA dependence and genetic rescue. We emphasize that identifying a Kpy-selective transferase would strengthen causal studies and might eventually provide a selective intervention point, while clearly stating that no such enzyme is currently known.

We thank the Editor and Referees again for their careful and constructive evaluation. We believe that the revised manuscript is more accurate, appropriately cautious and better focused on the experiments needed to establish the biological and translational significance of lysine pyruvylation.

Yours sincerely,

Jun Zhang, PhD

Shenzhen University Medical School

E-mail: junzh128@szu.edu.cn
ORCID: 0000-0002-6523-7573

18th Aug 2026

Dear Dr. Zhang,

We are pleased to inform you that your manuscript is accepted for publication and is now being sent to our publisher to be included in the next available issue of EMBO Molecular Medicine pending the final figure adjustments by our graphic designer, who will contact you for your approval of the figures.

Your manuscript will be processed for publication by EMBO Press. It will be copy edited and you will receive page proofs prior to publication. Please note that you will be contacted by Springer Nature Author Services to complete licensing information.

Should you be planning a Press Release on your article, please notify the production team via this form in order to coordinate publication and release dates: https://forms.cloud.microsoft/e/eH4A8JVP6g

If you have any questions, please do not hesitate to contact the Editorial Office. Thank you for your contribution to EMBO Molecular Medicine.

Yours sincerely,
Zeljko Durdevic

Zeljko Durdevic
Senior Editor
EMBO Molecular Medicine
